# Origins of cancer symposium 2015: posttranslational modifications and cancer

**Published:** 2015-07

**Authors:** Jason Cooper, Kevin Maupin, Nathan Merrill

**Affiliations:** ^1^ Van Andel Institute Graduate School, Grand Rapids, Michigan, USA

**Keywords:** cancer, posttranslational modifications, bioinformatics, mass spectrometry, review

## Abstract

The sixth annual Origins of Cancer Symposium was held July 10, 2015, at the Van Andel Research Institute in Grand Rapids, MI. Its theme was Beyond the Genome, with talks focused on the various influences of posttranslational modifications in malignant transformation and the spread of cancer. This event was organized by senior Ph.D. students as part of their professional development training at the Van Andel Institute Graduate School and is a successor to the original Oncogene meetings established by the Foundation for Advanced Cancer Studies in the 1980s. The symposium featured eight world-renowned scientists who disclosed their new findings and reflected upon past work related to the array of posttranslational modifications that contribute to cancer.

## INTRODUCTION

The cell is a magnificently organized and complex structure. Within each is a hierarchy of control that fine tunes the amount and activity of many thousands of unique proteins. For years the workflow of the cell seemed to be a straightforward process. From DNA, mRNA was synthesized and transported out of the nucleus. Once in the cytoplasm, these instructions could be read by enzymes that assembled functional proteins which could then do their work in and around the cell. However, this idea fails to capture the diversity of function and the scale of complexity that is truly at work. Posttranslational modifications (PTMs) are enzymatic modifications on proteins that are added during or after translational synthesis. The enzymes that control such changes are emerging as an important contributor to cancer [1]. The sixth annual Origins of Cancer Symposium dealt with PTMs and their varied roles in the formation and spread of cancer [2-4].

The symposium began with Dr. Sonia Lobo Planey (Commonwealth Medical College) highlighting the role of palmitoylation in cancer and interstitial cystitis. Her talk focused on the consequences of palmitoylation in signal transduction, with a specific focus on the signaling networks under the control of the *ZDHHC2* gene, which encodes a palmitoyltransferase implicated in cancer. Using a technique called palmitoyl-cysteine isolation capture and analysis, she identified substrates of ZDHHC2 in HeLa cells, including CKAP4/p63, a receptor for anti-proliferative factor (APF). APF is increased in patients suffering from interstitial cystitis [5]. Following up on this finding, she demonstrated that knock-down of ZDHHC2 inhibited the anti-proliferative activity of APF in cancerous and normal epithelium. Reduction in ZDHHC2 changed the distribution of CKAP4 from the endoplasmic reticulum to the perinuclear membrane [6]. With pharmacological modulation of palmitoylation, she concluded, the targeting of ZDHHC2 in cancer shows great promise.

The second speaker focused on the diverse functions of ubiquitination. Dr. Michael Rape (University of California, Berkley) discussed the broad array of ubiquitin chain formations and what these various chains mean for cell fate. He noted that aberrant processes of ubiquitination have been implicated in cancer, neurodegeneration, and rare developmental diseases [7]. He communicated to the audience soon-to-be published work that used stem cell models to elucidate the role of the cullin-based ubiquitin ligase (CUL3) in the formation of appropriate embryo neuron morphology, saying that this enzyme “is a switch that sits between neural fates.” Loss of CUL3 activity leads to the loss of all neural crest cells because their precursors erroneously become forebrain precursors. Using a novel ubiquitin substrate screening pipeline called ComPASS to search for unknown proteins interacting with CUL3, he found that TCOF1 and NOLC1 (scaffolds for ribosome biogenesis) were top candidates. Through these proteins, CUL3 controlled not ribosome biogenesis itself but the output of ribosomes during different stages of development. Dr. Rape suggested that, using this research as a starting point, the activity and output of ribosomes may someday be controlled in the laboratory.

Dr. Scott Coonrod (Cornell University Weill Medical College) gave a talk on peptidylarginine deiminase 2 (PAD2) and its role in the PTM called citrullination, which is the conversion of arginine into a citrulline. PADs have a role in female fertility due to their control of oocyte cytoskeletal formation [8]. The deregulation of PAD enzymes is involved in cancers and autoimmune disorders [9]. Dr. Coonrod's lab has shown that PAD2 expression is up-regulated in tamoxifen-resistant breast cancer cells [10]. While they predicted that PAD2 might be a driver of breast cancer, unexpectedly, overexpression of PAD2 in mice by the murine mammary tumor virus promoter led to dysplastic tumors in skin cells, but not in mammary glands [11]. This work suggested that PAD2-driven oncogenesis in the skin was due to increased production of inflammatory cytokines, which were not observed in the mammary epithelium. Future work will focus on observing the effects of PAD inhibitors in breast cancer. Studies have shown that the inhibitor BB-Cl-amidine blocked cell growth and increased apoptosis in breast cancer cells but not in normal cells. Taken together, these results suggest that while PAD2 can be a tumor promoter in some cell types, it may be more important in later stages of breast cancer.

Dr. J. Michael Pierce (University of Georgia Cancer Center) gave a “universal” introduction to demonstrate how abundant carbohydrates truly are, explaining how simple carbohydrate compounds have been detected within interstellar dust clouds [12]. He went on to show the many ways that cells have adapted carbohydrates for functional use through the covalent attachment of oligosaccharides to proteins and lipids (glycosylation). He also discussed how changes in cell surface glycosylation are a hallmark of oncogenesis [13, 14]. Dr. Pierce's research has focused on the role of the glycosyl transferase, Gnt-V, in supporting oncogenic growth factor signaling. This is accomplished through increased *N*-glycan branching and subsequently increased retention of growth factor receptors on the cell surface [15-17]. His data suggest that therapeutically targeting Gnt-V might lower tumor cell proliferation and survival by reducing the availability of several growth factor receptors at the cell surface.

Tony Hunter (Salk Institute) began his talk by emphasizing the importance of PTMs in drastically increasing the number of possible unique proteoforms and their associated activities. While the human genome has approximately 20,000 genes, alternative splicing of mRNA brings the number of possible transcripts to an estimated 100,000. PTMs can further increase the number of proteoform possibilities to well over 1,000,000 [18]. Dr. Hunter then highlighted the events leading to the “accidental” discovery of tyrosine phosphorylation, which has led to 28 clinically approved kinase inhibitors over the past 30 years [19, 20]. Turning to recent work, Dr. Hunter emphasized the importance of cancer cell interactions with the microenvironment. He detailed how, via a feedback loop, pancreatic cancer cells can signal to stellate cells through platelet-derived growth factor, which produces a complex signaling cascade back to the cancer cells [21]. In closing, Dr. Hunter emphasized to younger scientists to never discount any results, because what seems like a mistake could end up opening an entire new field of discovery.

Dr. Mauricio Reginato (Drexel University College of Medicine) is interested in understanding how oncogenes regulate cancer cell signaling and how they alter metabolic reprogramming. He discussed how the posttranslational modification of proteins by the monosaccharide *N*-acetylglucosamine (*O*-GlcNAcylation) orchestrates cell signaling to reflect the nutrient status of the cell [22]. Dr. Reginato showed that *O*-GlcNAcylation is up-regulated in cancer and that this modification is a driver of aerobic glycolysis by promoting the stabilization of HIF-1α [23-25]. This happens through a reduction in total 2-oxoglutarate, a co-factor required for HIF-1α prolyl-hydroxylation, and HIF-1α's subsequent targeting for degradation by the VHL E3 ubiquitin ligase [26]. His results underline the importance of this signaling pathway in metabolism and suggest that the *O*-GlcNAc transferase could be an effective therapeutic target in highly glycolytic tumors.

Dr. Karolin Luger (Colorado State University) began her presentation by showing how many of the common histone modifications that are known to influence gene expression (lysine methylation and acetylation) have very little effect on nucleosome structure [27]. Thus, the major function of these epigenetic marks is not to alter histone DNA interactions directly, but to recruit the proteins that bind these modifications [28]. Dr. Luger went on to show her most recent data on the role of the poly ADP-ribose polymerase 1 (Parp1) in nucleosome assembly. In addition to Parp1's well known role in DNA damage and repair, her studies revealed that Parp1 auto-PARylation alters Parp1's affinity for intact chromatin and leads to the efficient assembly of new nucleosomes [29, 30]. These results suggest that events that trigger Parp1 auto-PARylation (e.g., DNA damage) can trigger Parp1's histone chaperone activity, and provide a better understanding of what Parp inhibitors do therapeutically.

The closing speaker, Dr. Michael Freitas (Ohio State University), detailed the evolution of mass spectrometry (MS)-based proteomics and how this is being used to study PTMs in cancer [31]. Proteomics has undergone a shift: the most time-consuming phase of a study is no longer sample preparation, but rather data analysis. Even with the rapid evolution of bioinformatics software, there are massive amounts of data left unscrutinized after MS because we do not know how to interpret it. His laboratory focuses on linker histone PTMs, which have been found to be crucial for histone organization and cell survival [32, 33]. His lab identified a phosphorylation site on histone H1 that is a better marker of tumor grade and proliferation in bladder cancer than traditional markers such as Ki-67 [34]. The challenge moving forward is determining how these histone modifications are altering cancer cell behavior and whether they can be targeted therapeutically.

With the rapid technological advances in analytical tools (e.g., MS fragmentation methods, glycan-binding reagents, and bioinformatics), it is becoming easier to identify and study novel PTMs [13, 35-37]. A better understanding of PTMs, the enzymes that add or remove them, and their functional impact will lead to the development of novel drug therapies and/or biomarkers for diseases in which a particular PTM is aberrantly regulated. While much of the focus in cancer has been on targeting kinase phosphorylation, this year's Origins of Cancer speakers highlighted the diversity of enzymes and PTMs that support tumorigenesis, opening a door to additional drug and biomarker discovery [38]. With some 1,000,000 proteoforms to study, it will be challenging to determine how the PTMs on a given protein interact to dictate that protein's function [18]. However, this means that there is plenty of research yet to be performed, and that is very exciting.
